# Confirmation of significant sea turtle nesting activity on a remote island chain in the Gulf of Mexico

**DOI:** 10.1002/ece3.10448

**Published:** 2023-08-21

**Authors:** Margaret M. Lamont, Dianne Ingram, Todd Baker, Matt Weigel, Brian M. Shamblin

**Affiliations:** ^1^ Wetland and Aquatic Research Center U.S. Geological Survey Gainesville Florida USA; ^2^ Deepwater Horizon Gulf Restoration Office U.S. Fish and Wildlife Service Fairhope Alabama USA; ^3^ Louisiana Coastal Protection and Restoration Authority Baton Rouge Louisiana USA; ^4^ Louisiana Department of Wildlife and Fisheries Baton Rouge Louisiana USA; ^5^ Warnell School of Forestry and Natural Resources University of Georgia Athens Georgia USA

**Keywords:** aerial survey, Gulf of Mexico, Kemp's ridley, sea level rise, sea turtle

## Abstract

Globally, six of the seven sea turtle species are threatened or endangered and as such, monitoring reproductive activity for these species is necessary for effective population recovery. Remote beaches provide a challenge to conducting these surveys, which often results in data gaps that can hamper management planning. Throughout the summer of 2022, aerial surveys were conducted over the Chandeleur Islands in the Gulf of Mexico. Turtle crawls were photographed for subsequent review by 10 expert observers. Whenever possible, ground surveys were conducted, and samples of unhatched eggs or dead hatchlings were collected. A summary of historic reports of sea turtle nesting activity at this site was also compiled. On 11 days between May 4, 2022, and July 30, 2022, photographs of 55 potential sea turtle crawls were taken. Observers identified 54 of those as being made by a sea turtle. There was high‐to‐moderate certainty that 16 of those crawls were nests, that 14 were made by loggerheads, and that two were made by Kemp's ridleys. Observers were least certain of species identification when surveys were conducted during rainy weather. Genetic analyses based on mitochondrial and nuclear DNA were conducted on samples from five nests and those analyses confirmed that three nests were laid by Kemp's ridleys and two were laid by loggerheads. Historic records from the Chandeleur Islands substantiate claims that the Chandeleurs have supported sea turtle nesting activity for decades; however, the consistency of this activity remains unknown. Our aerial surveys, particularly when coupled with imaging, were a useful tool for documenting nesting activity on these remote islands. Future monitoring programs at this site could benefit from a standardized aerial survey program with a seaplane so trends in nesting activity could be determined particularly as the beach undergoes restoration.

## Graphical abstract

Remote beaches provide a challenge to conducting surveys for nesting sea turtles, which often results in data gaps that can hamper management planning. Aerial imaging surveys over the Chandeleur Islands, Louisiana, resulted in the documentation of 54 sea turtle crawls over 11 days in 2022, which represents relatively dense nesting activity for the northern Gulf of Mexico.

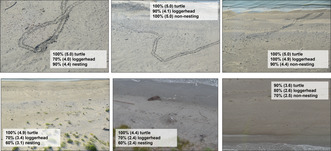

## 1 INTRODUCTION

Surveys for sea turtle nesting activity occur on beaches throughout the world, including along the Gulf of Mexico coast, and these data allow monitoring of population trends (Ceriani et al., 2019). Four sea turtle species nest on Gulf coast beaches including the loggerhead (*Caretta caretta*), Kemp's ridley (*Lepidochelys kempii*), green (*Chelonia mydas*), and leatherback (*Dermochelys coriacea*). This region is particularly significant in that it supports the primary nesting beaches for critically endangered Kemp's ridleys (Shaver et al., 2016) and provides nesting habitat for the two smallest, genetically unique Recovery Units (RU) of Western Atlantic loggerheads: the Dry Tortugas Recovery Unit that averaged 246 nests from 1989 to 2007 and the Northern Gulf of Mexico Recovery Unit that averaged 906 nests during that same time span (NMFS & USFWS, 2008; Richards et al., 2011). The small sizes of these RUs make them vulnerable to population extinction (Legendre et al., 1999; Shaffer, 1981; Traill et al., 2010).

In addition to increased vulnerability due to small population sizes, sea turtles nesting on Gulf of Mexico beaches also face pressures from commercial fishing activities, anthropogenic and natural debris (Fujisaki & Lamont, 2016), and intensive energy activities (Hart et al., 2018); those activities resulted in one of the largest oil spills in US history, which occurred in the northern Gulf of Mexico in April 2010 (Deepwater Horizon; Bjorndal et al., 2011). Additionally, much of the Gulf of Mexico is fringed by low‐lying barrier islands that are highly susceptible to erosion and inundation (Lamont et al., 2012; Ware & Fuentes, 2020). These coastal dynamics may drive shifts in sea turtle nesting distribution (Fujisaki et al., 2018; Lamont & Houser, 2014), which may complicate sea turtle monitoring efforts. Recent evaluation of nesting trends in the Northwest Atlantic loggerhead population found no recovery, and a clear understanding of why recovery is not occurring (or has not been documented) is lacking (Ceriani et al., 2019). Similar trends in Kemp's ridley nesting have been documented. After decades of apparent recovery, Kemp's ridley nesting declined significantly in 2010 and full recovery from that decline remains unclear as do the reasons for the declines (Galloway et al., 2016; Reyes‐López et al., 2021). Tagging studies in the northern Gulf of Mexico showed that these loggerheads exhibited extremely low nesting site fidelity (Hart et al., 2014; Lamont et al., 2014). This low fidelity may represent exploratory behavior, which has also been suggested for loggerheads that are expanding their nesting range in the Mediterranean (Hochscheid et al., 2022).

Sea turtle nesting is currently monitored on hundreds of beaches across the Gulf of Mexico. The Florida Fish and Wildlife Conservation Commission (FWC), in partnership with the US Fish and Wildlife Service (USFWS), oversees monitoring programs on 215 beaches along the Florida coast that encompass 1328 km of habitat (https://myfwc.com/research/wildlife/sea‐turtles/nesting/monitoring/). Similar surveys in Alabama, Mississippi, and parts of Texas are overseen by the USFWS, whereas in southern Texas, NPS biologists at PAIS monitor nesting sea turtle activity (Shaver et al., 2016). Gaps in monitoring occur where logistics make surveying difficult and/or where sea turtle nesting habitat is not available (e.g., Big Bend region of Florida; Hart et al., 2020). Remote beaches often represent the largest gaps in nest monitoring due to significant challenges associated with accessing those sites, and yet these remote sites may also include important nesting locations (Flores et al., 2021; Khan et al., 2010). Identifying these sites and establishing standardized monitoring at dense nesting locations will help managers better understand the fluctuations in nesting activity.

**FIGURE 1:**
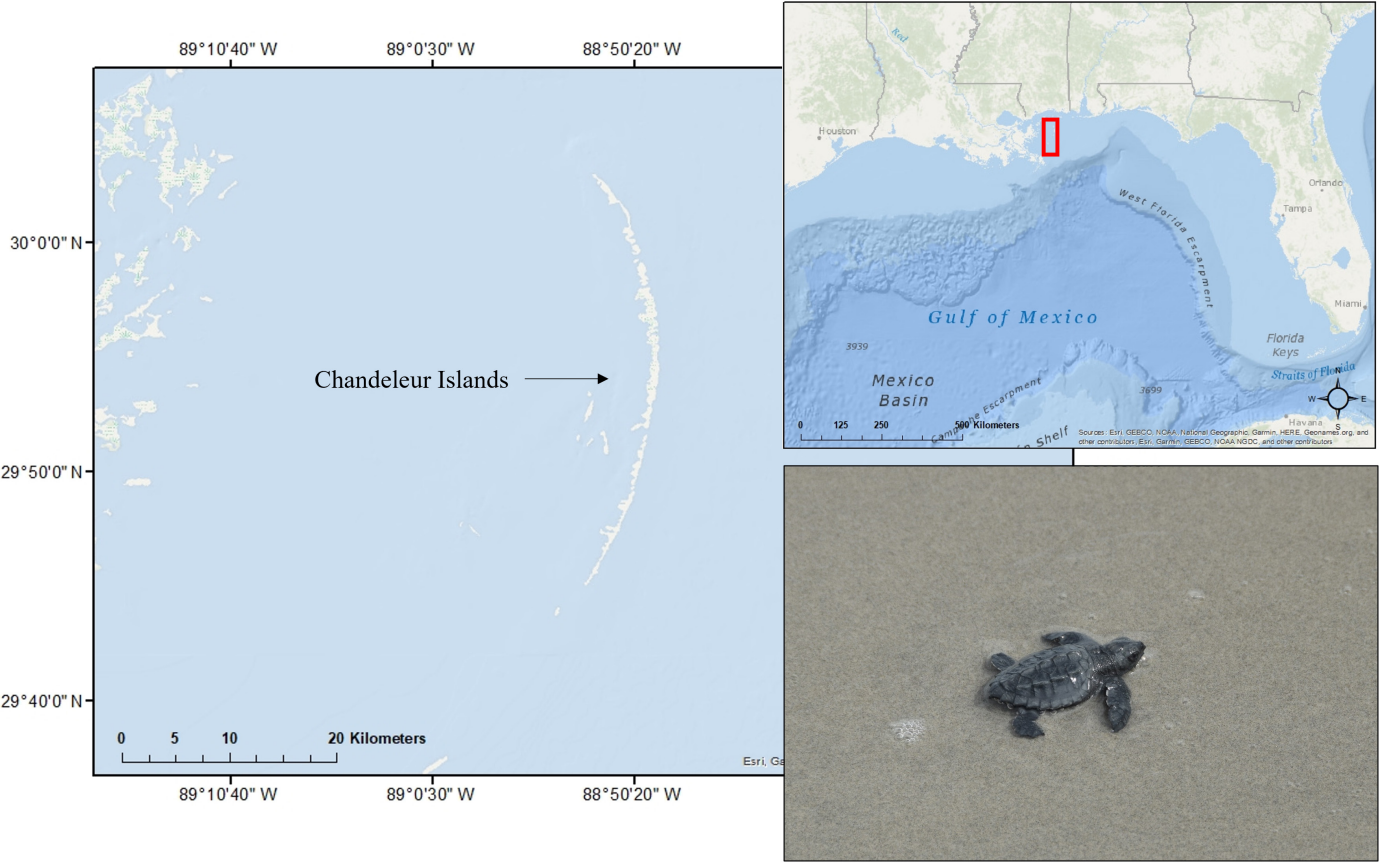
Chandeleur Islands lie off the eastern coast of Louisiana in the northern Gulf of Mexico (red box in inset). Several Kemp's ridley hatchlings (including the one photographed here by M. Weigel) were observed on the beach after emerging from a nest on the Chandeleur Islands on July 29, 2022. One dead hatchling was also found, and genetic analysis confirmed the species identification.

The Chandeleur Islands are a chain of low‐lying barrier islands that lie approximately 70 km off the coast of southeastern Louisiana in the northern Gulf of Mexico (Figure 1). Approximately 35 km in length, the entire island chain is protected habitat and falls within the boundaries of Breton National Wildlife Refuge. The islands are difficult to access and traverse and as such no formal surveys have been conducted for nesting sea turtles on the islands. Opportunistic sightings of sea turtle nesting activity have previously been reported; however, details of this previous nesting activity, such as species identification, have been lacking (Ogren et al., 1978). In this project, we compiled reports of previous sea turtle activity on the Chandeleur Islands and conducted 1‐year of aerial surveys, with opportunistic ground‐truthing of observed crawls, to document sea turtle nesting activity along this remote island chain.

## 2 METHODS

### 2.1 Summary of previous nesting activity

We contacted biologists and natural resource managers at multiple agencies across the region requesting any information they had about sea turtle nesting activity on the Chandeleur Islands or elsewhere in Louisiana. We were seeking both formal (e.g., published literature) and informal (e.g., emails) information. The agencies we contacted included: Louisiana Department of Wildlife and Fisheries (LDWF); US Fish and Wildlife Service, Breton National Wildlife Refuge, Louisiana Ecological Services Field Office, and Gulf Restoration Office; National Marine Fisheries Service, Audubon Aquarium, and Padre Island National Seashore. Additionally, we used Google Scholar with the following search terms: Chandeleur, Louisiana, Breton, sea turtle, turtle, Kemp's ridley, loggerhead, *L. kempii*, *Ca. caretta*, nest, nesting, crawl, to locate additional reports. All documents and mentions of sea turtle activity on the Chandeleur Islands were retained including the unpublished literature, theses and dissertations, reports, and published literature.

### 2.2 Aerial surveys and ground‐truthing

Using either a Kodiak 100 or Cessna 206 seaplane, aerial surveys were flown along a 29 km stretch of the Chandeleur Islands, Louisiana from May 27, 2022, to August 15, 2022. The entire survey beach falls within the boundaries of Breton National Wildlife Refuge. All surveys were flown relatively low (at approximately 37 m under a special use permit #43556‐22‐02; Epperly et al., 1995, Fuentes et al., 2015) to ensure image resolution was clear enough for species identification, at a speed of 80–90 kts (Fuentes et al., 2015). Because most sea turtles nest at night (except Kemp's ridleys; see Shaver et al., 2016), surveys were initiated early in the morning (typically before 7 a.m.), except when poor weather conditions forced delayed take‐off, so any existing turtle crawls were fresh and unaffected by weather (e.g., wind, rain; Crouse, 1984). In addition to the pilot, two biologists flew in the aircraft, one in the right front passenger seat and one in the rear seat immediately behind the front passenger. When a suspected crawl was observed, the aircraft circled the area, a GPS coordinate was collected, and biologists took handheld photographs through the aircraft window with a Nikon D3200 camera with an AF‐S Nikkor 18‐55 mm lens or a Nikon AF‐S DX camera with a Nikkor 18‐200 mm lens. During each flight, biologists compared new crawls to data and GPS locations for previously documented crawls to ensure crawls were not recorded twice.

In‐flight documentation of crawls included information on the flight (e.g., altitude and travel speed), crew and location and a GPS coordinate for the crawl. Whenever possible, the seaplane would land, and biologists would walk to the crawl site for ground‐truthing. On‐the‐ground documentation of crawls included a more precise GPS coordinate for the crawl/body pit, and measurements of crawl width, length, distance to high tide line, and distance to dune or vegetation. These measurements are generally species‐specific (FWC, 2016; Stapleton & Eckert, 2008) and provide information about nest site selection that can help guide beach restoration activities (Wood & Bjorndal, 2000).

Finally, during ground‐truthing trips, suspected turtle nests were observed whenever possible to document hatchling emergence, and nest excavations were conducted on a subsample of nests after the incubation period was completed (defined as 75 days after the first day the crawl was documented) to document emergence success (defined as # of hatched eggs subtracted by the total number of live and dead hatchlings in the nest all divided by the total # of eggs; Johnson et al., 1996). If any salvage materials, including undeveloped eggs, embryos, hatched eggshells or dead hatchlings were encountered, biologists placed some combination of those samples in a clean plastic bag and stored them in a freezer for genetic analyses.

### 2.3 Genetic analyses

Samples collected from sea turtle nests laid on the Chandeleurs were sent to the University of Georgia for species identification and maternal relatedness. DNA was extracted from embryos and eggshells using a modified DNeasy protocol as previously described (Shamblin et al., 2011). DNA from the least degraded sample from each nest based on visual inspection was used to generate a mitochondrial control region haplotype to confirm maternal species. PCR used primers LCM15382 and H950g (Abreu‐Grobois et al., 2006) and sequencing primers LCM15382 and an internal primer, CC4413 as previously described (Shamblin et al., 2012). Sequences were compared with standard nomenclature developed for Kemp's ridleys by NOAA Southwest Fisheries Science Center Sea Turtle Genetics Program and for loggerheads by the Archie Carr Center for Sea Turtle Research (https://accstr.ufl.edu/resources/mtdna‐sequences/). Genotyping at 16 nuclear microsatellite loci was attempted for all collected samples using three multiplex reactions as described for loggerhead eggshells from the Northern Recovery Unit (Shamblin et al., 2021). All loci were originally isolated from loggerheads (Shamblin et al., 2007), and five are diagnostic in distinguishing Kemp's ridleys from loggerheads based on nonoverlapping allele sizes (Shamblin et al., 2009; B. M. Shamblin, unpublished data).

### 2.4 Observer characterizations

Aerial photographs were compiled and shared with expert image observers. These experts were selected by the USFWS as having experience observing loggerhead, Kemp's ridley, and green turtle crawls and nests as they were the species most expected to be encountered on the Chandeleur Islands. Each observer reviewed each photograph and recorded whether the crawl had been made by a sea turtle, and if so, identified the species and whether it was a nest or a non‐nesting emergence. Observers also ranked each characterization for certainty on a scale from one to five. Ranking categories were as follows: (1) strong uncertainty, (2) moderate uncertainty, (3) unknown certainty/uncertainty, (4) moderate certainty, and (5) strong certainty. For example, if an observer was extremely certain that a crawl was made by a sea turtle they would enter “yes” and rank their certainty as a five. However, if the photograph of the crawl was blurred (e.g., by rain) making species identification challenging, the observer would select their best guess as to which species made the crawl and rank their certainty as a one. Observers evaluated the photographs without reviewing the information gathered during ground‐truthing.

The overall proportion of characterizations (e.g., crawl identification and nest/non‐nesting emergence) was calculated along a mean rank of certainty for each characterization. Rankings were categorized into three groups: (1) high included rankings 4 and 5, (2) moderate included ranking 3, and (3) low included rankings 1 and 2.

We calculated crawl density (number of emergences/km), number of crawls per day and crawl density per day to compare nesting activity on the Chandeleur Islands to that reported to the Florida Fish and Wildlife Conservation Commission (FWC) for the seven counties in Northwest Florida where Statewide Nesting Beach Surveys are conducted for the northern Gulf of Mexico loggerhead group. For daily calculations, we assumed surveys on Northwest Florida beaches occurred every day from May 1 to August 31 (123 days), which is the time period suggested by FWC (https://myfwc.com/media/3133/fwc‐mtconservationhandbook.pdf).

## 3 RESULTS

### 3.1 Summary of previous nesting activity

Twenty documents that mentioned previous sea turtle nesting activity on the Chandeleur Islands, LA, were gathered. The documents ranged from personal observations of sea turtles or sea turtle crawls provided to natural resource managers (e.g., NOAA stranding coordinators or LDWF biologists) to published papers. The earliest reference to Kemp's ridley nesting on the Chandeleur Islands was the 1961 article by Percy Viosca in the *LDWF Conservationist* entitled “Turtles, tame and truculent.” Documentation of actual survey effort on the island was in a report to the National Marine Fisheries Service in 1977. The most recent reporting of sea turtle nesting on the Chandeleur Islands was an email correspondence and video sent to NMFS from a fisherman who observed a nesting Kemp's ridley on the islands in 2018. Sea turtle nesting at other sites in Louisiana, particularly Grand Isle, was also reported with one documented nest as early as the 1930s (Hildebrand, 1979). Although these communications provide information on previous nesting activity along the Chandeleur Islands, very few details about those turtles are available. For example, aside from the one report that included video evidence of a Kemp's ridley nesting on the Chandeleurs, no other reports of sea turtle nesting activity specified turtle species. Additionally, these previous reports typically lacked clarification about whether observed crawls were nesting or non‐nesting emergences and no information on hatching success was provided.

### 3.2 Aerial surveys

On 11 days between May 4, 2022, and August 15, 2022, photographs of 55 potential sea turtle crawls were gathered (Tables 1 and 2). The first crawl was located and photographed on May 4, 2022, by Robert Dobbs an Ornithologist with LDWF who was on the island conducting surveys for shorebirds. Aerial surveys began on May 27, 2022, and were conducted on 10 days total. Of those 10 aerial survey days, crawls were documented on 8 days (average 5.3 crawls/day), with peak numbers observed on June 15 (*n* = 13) and June 24 (*n* = 15). Overall crawl density (i.e., # crawls/length of beach) on this 29 km stretch of beach was 1.9 crawls/km, which is similar to crawl density documented on Northwest Florida and Alabama beaches (Table 3; https://myfwc.com/research/wildlife/sea‐turtles/nesting/statewide/, unpublished USFWS data). Chandeleur Islands had the third greatest number of crawls per day compared with Northwest Florida and Alabama, and when length of survey beach was also accounted for along with number of survey days, the Chandeleur Islands had the second greatest crawl density in this region (0.19 crawls/km/day).

**TABLE 1:** List of days when aerial surveys were conducted over the Chandeleur Islands, Louisiana.

Survey date	Total survey time (min)	# Crawls
May 27, 2022	44	2
June 3, 2022	55	8
June 8, 2022	40	3
June 15, 2022	57	13
June 24, 2022	67	17
June 30, 2022	56	8
July 22, 2022	39	1
July 29, 2022	30	1
August 5, 2022	29	0
August 15, 2022	14	0

*Note*: A sea turtle crawl was also documented on May 4, 2022 by a Louisiana Department of Fisheries and Wildlife biologist conducting shorebird surveys.

**TABLE 2:** Sea turtle crawls per kilometer per day across all counties in Northwest Florida and Alabama and along the Chandeleur Islands, LA in 2022 and mean data for all crawls documented on the Chandeleur Islands, Louisiana (in italics) during 10 aerial survey flights conducted from May to July 2022.

County	Survey length (km)	Crawls/km/day
Franklin	98.0	0.128
Gulf	47.0	0.273
Bay	71.1	0.041
Walton	48.7	0.019
Okaloosa	38.0	0.013
Santa Rosa	11.2	0.020
Escambia	64.8	0.024
Alabama	74.8	0.018
Chandeleurs	*29.0*	*0.186*
	**Cc**	**Lk**
Crawl width (cm)	79.4	64.3
Crawl length (cm)	37.5	51.1
Apex to high tide (cm)	20.3	41.9
Apex to dune (cm)	9.7	9.5
% Certainty	78.3	85.0

*Note*: Apex to high tide and apex to dune—distance from the highest point of the crawl to the high tide or dune; % certainty—the mean certainty ranking of the species identification from all 10 image observers. Lk—Kemp's ridley; Cc—loggerhead.

**TABLE 3:** Hatching information for two nests (one found on June 24 and the other on July 29) laid on the Chandeleur Islands, LA that were excavated after incubation was complete and all viable hatchlings had emerged.

	6_24_22_15	7_29_22_01
Total # hatched eggs	76	0
# Live hatchlings	0	0
# Dead hatchlings	0	0
# Hatchlings emerged	76	0
Total # pipped eggs	0	0
# Of pipped eggs with live embryos	0	0
# Of pipped eggs with dead embryos	0	0
Total # of unhatched eggs	24	139
Total # of eggs in nest	100	139

**FIGURE 2:**
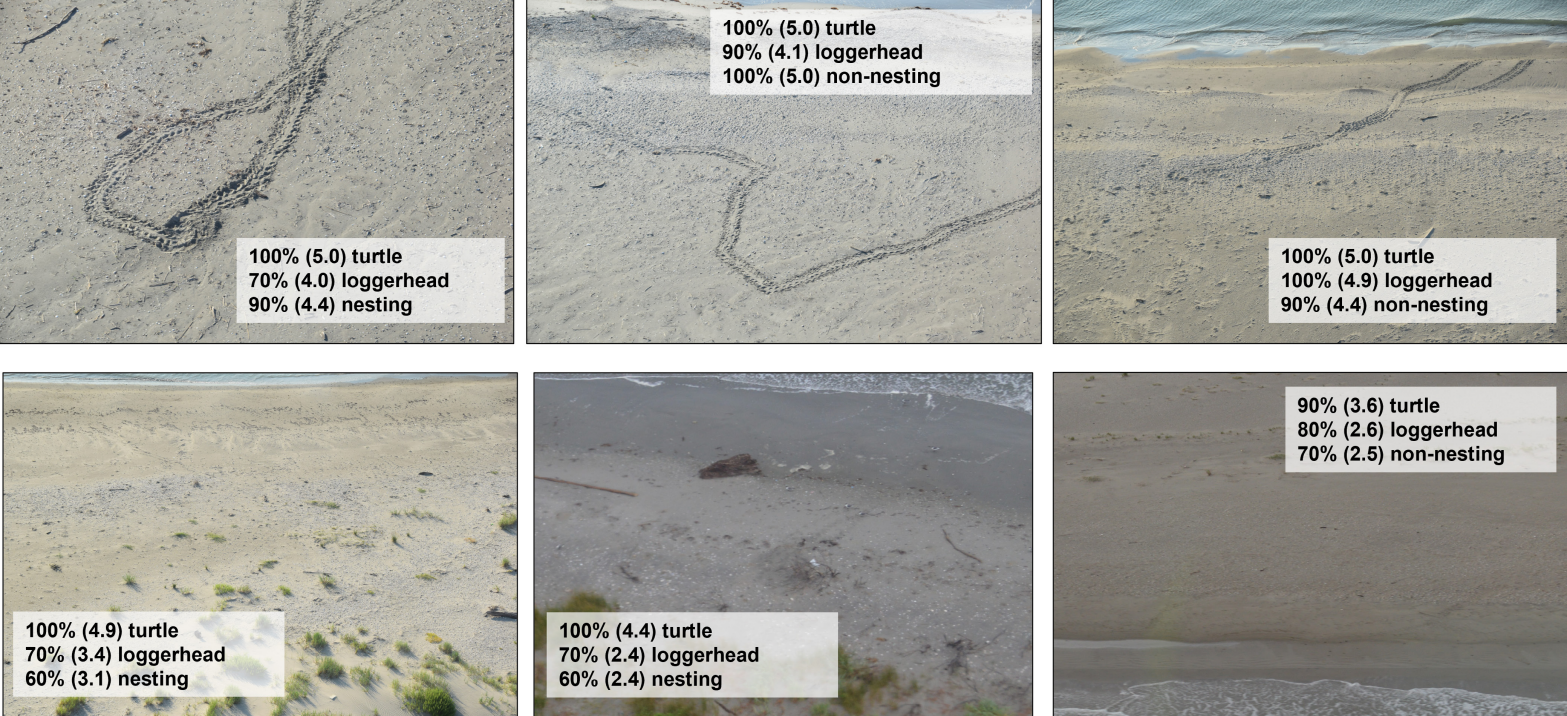
Examples of photographs taken from the aircraft of sea turtle crawls on the Chandeleur Islands, LA, in summer 2022. Images taken in clear sunny weather (a) resulted in more confident identifications by reviewers than those taken in rainy weather (b). The proportion of reviewers who agreed with each characterization along with the mean observer certainty rankings (in parentheses) are included on each image: was the crawl made by a sea turtle, what species of sea turtle made the crawl, and was it a nesting or non‐nesting crawl.

Of all 55 crawls, biologists were able to ground‐truth 15 of them, confirm that five were indeed nests and gather nest content samples from all five of those nests after hatching was completed and all viable hatchlings had emerged. Full nest excavations were only conducted on two of those five nests due to logistical challenges (Table 4). Samples from all five nests included the carcass of one dead hatchling, dead embryos from four unhatched eggs, seven unhatched eggs at unidentified stages of development, and eggshells from two hatched eggs. Biologists also documented several live hatchlings emerging from the second nest found on July 29, 2022 (Figure 2).

**TABLE 4:** Number of sea turtle crawls, nests or non‐nesting emergences per species with observer certainty categories (high, medium, low).

	Sea turtle crawl		Nest/ non‐nesting emergences		Species ID	
	Yes	No	Nest	FC	Loggerhead	Kemp's
High	46	1	5	18	15	2
Medium	7	0	4	3	15	2
Low	0	0	9	14	20	0

Crawl data collected from 14 crawls of the 15 crawls that were ground‐truthed (one crawl was found opportunistically and as such crawl data were not collected) included 10 crawls identified by observers as loggerheads and four as Kemp's ridleys. Of those, 14 crawls genetic analyses confirmed two were made by Kemp's ridleys and two were made by loggerheads (all four were correctly identified by observers). Loggerhead crawls were larger than Kemp's ridley crawls, were generally shorter in length and did not extend as far above the daily high tide line (Table 2).

Of all 55 crawls, observers characterized 54 as being made by a sea turtle (mean certainty 4.7) and one as unidentified (mean certainty 2.4; Table 4). Of those 54 crawls, observers had high certainty (certainty ranking 4 or 5) that 45 were made by a sea turtle and moderate certainty (certainty ranking 3) that the remaining seven crawls were made by a sea turtle.

Of those 54 crawls characterized as being made by a sea turtle, observers had high certainty that five were nests and moderate certainty that nine were nests (Table 4). There was high certainty that 18 were non‐nesting emergences and moderate certainty that 21 were non‐nesting emergences. Observers had low certainty that the remaining 13 crawls were nests or non‐nesting emergences. Mean overall certainty of nest characterizations was 3.2 and non‐nesting emergence characterizations was 3.5. Image observers agreed with observers in the aircraft as to whether crawls were nesting or non‐nesting emergences on all but four crawls (92.7% consensus). Of the 15 crawls that were ground‐truthed, image observers agreed with biologists on the ground as to whether a crawl was a nesting or non‐nesting emergence on 14 (93.3%) of those crawls.

Observers had high certainty that 14 crawls were made by loggerheads and two were made by Kemp's ridleys, and moderate certainty that 13 were made by loggerheads and two were made by Kemp's ridleys. They had low certainty about the species identification of the remaining 20 crawls. Mean overall observer certainty of species identifications was 3.3.

Observers were least certain of species identification when surveys were conducted on rainy days (June 30, July 22), whereas observer certainty was moderate to high on days that were sunny or partly cloudy (Figure 2). However, this pattern was not as evident in observer certainty of nesting activity. Certainty was lowest during rain but was also low on two partly cloudy days where corresponding species identification certainty was high (May 27) or moderate (June 3).

### 3.3 Genetic analyses

Genetic analyses based on mitochondrial and nuclear DNA confirmed the species of three nests as Kemp's ridley and two as loggerhead. Species diagnostic and informative microsatellite alleles indicated that dead embryos and dead hatchlings represented pure species with no indication of hybridization.

The Kemp's ridley embryo from the June 24 nest was too badly degraded to provide complete microsatellite data, but all three Kemp's ridley nests (June 8, June 24, July 29_02) yielded control region haplotype Lk1.1 (Genbank KF385935). This haplotype is quite rare in the Texas nesting population (2%, Frandsen et al., 2020) and among foraging individuals (4%, Frandsen et al., 2020; 2%, Lamont et al., 2021), suggesting that all three clutches were likely laid by the same female.

The two loggerhead clutches (June 15, July 29_01) were assigned to the same female. Genotypes from three undeveloped eggs from the nest documented on June 15 directly matched, indicating that they were maternal. This genotype was compared with a database of nesting females sampled in Alabama and northwestern Florida but did not match any known individuals. A dead embryo from nest July 29_01 was determined to be an offspring of this female based on parentage analysis. She carried control region haplotype CC‐A1.1 (Genbank EU179436), the most common variant in the Northern Gulf of Mexico Recovery Unit (Shamblin et al., 2012).

Of those nests where species was confirmed genetically, observers correctly identified species from photographs on 4 of the 5 emergences. Of the four crawls where observers correctly identified the species, the mean certainty rankings ranged from 3.6 to 4.5 (overall mean 4.2). The one nest that was incorrectly characterized by observers was genetically identified as being deposited by a Kemp's ridley. The three observers that correctly identified the crawl as a being made by a Kemp's ridley were confident in their characterization with a mean certainty ranking of 4.3 whereas the 7 observers who mis‐identified the crawl as being made by a loggerhead were less certain in their characterization (mean ranking 2.4).

## 4 DISCUSSION

Historic records suggest sea turtles have nested on the Chandeleur Islands, LA, for decades (Ogren, 1989). This location appears to serve as a nesting site for both threatened loggerheads and endangered Kemp's ridleys. Genetic analyses confirmed use of this barrier island chain by both species and established within‐season fidelity to this beach by at least one female loggerhead. Including photographs of crawls in our aerial surveys allowed multiple observers to view crawls and add certainty rankings to their characterizations, which provided managers with more detailed results. These findings further support the idea that remote beaches play an important role in maintaining sea turtle populations, particularly during periods of environmental instability (Thomas et al., 2012).

In the 1940s, as many as 10 turtles of unspecified species were reported nesting in one night on the Chandeleur Islands (Ogren et al., 1978) and in 1962, 32 crawls (also unidentified species) were documented during one aerial survey of the island chain (Ogren et al., 1978). Loggerhead nesting in Florida increased from the late 1970s through the late 1990s before declining for approximately 10 years and then rebounding to 1990s levels starting around 2009 (Ceriani et al., 2019; Conley & Hoffman, 1987). Our daily crawl density aligns with other nesting sites in Northwest Florida that primarily support the genetically distinct Northern Gulf of Mexico nesting subpopulation of loggerheads (Ceriani et al., 2019).

Our review of historic nesting activity on the Chandeleur Islands substantiates claims that the Chandeleurs have supported sea turtle nesting activity for decades; however, the consistency of this activity remains unknown. Questions remain such as: (1) Do the Chandeleur Islands represent a consistent nesting site for loggerheads and Kemp's ridley or are they fringe habitat on the edge of the primary nesting sites? (2) Has nesting density remained consistent over the past century but just gone undocumented or has nesting density fluctuated? If it has fluctuated, is it due to changes in sea turtle population numbers, range expansion or something else? (3) What is the within‐ and among‐species genetic connectivity of loggerheads and Kemp's ridleys nesting on the Chandeleur Islands? And are there multiple individuals using these beaches or only a few that emerge repeatedly?

Aerial surveying is a useful tool for documenting sea turtle nesting activity, particularly on remote beaches (Crouse, 1984; Dunstan et al., 2020; Rees et al., 2018; Witt et al., 2009). The addition of imaging capabilities greatly increased our ability to assess species identification and nesting activity (Dunstan et al., 2020). Observer certainty was reduced when surveys were conducted during or immediately following rain; the age of the crawl and wind‐blown beaches also appeared to reduce certainty particularly in identifying nesting activity. As such we recommend future surveys target dry, clear days and ground‐truthing occur as often as possible to confirm nesting activity. Ground‐truthing was beneficial in multiple ways including allowing close‐up photographs and allowing for collection of samples for genetic analyses. Future monitoring programs at this site could benefit from a standardized aerial survey program with a seaplane so trends in nesting activity could be determined particularly as restoration planning for this beach is conducted (see ap24.coastal.la.gov/projects/chandeleur‐islands).

Over the past two decades, Kemp's ridleys appear to have expanded their nesting range with sporadic nesting documented along the Gulf coast into Florida (Pike, 2013; Shaver et al., 2016). Recent genetic analyses also suggest the species may be undergoing range expansion (Frandsen et al., 2020; Lamont et al., 2021). As species undergo range expansion across the globe (Henry III et al., 2021), protected areas are being disproportionally settled (Thomas et al., 2012). The Chandeleur Islands fall within the Breton National Wildlife Refuge and as such benefit from National Wilderness System status (https://www.fws.gov/refuge/breton). This, along with their remote location, provides significant protection to the habitats and species using the islands. While the Refuge is open to visitors, it is only open during daylight hours, no camping is allowed, no fires are permitted, and damaging vegetation in any way is not allowed. Recreational fishing and crabbing are permitted in the surrounding waters; the use of nets and trotlines and commercial fishing is not allowed. Important habitat for nesting and wintering shorebirds is designated and visitors are not permitted to enter those areas. Additionally, many threats to nesting turtles often found on populated beaches such as artificial lighting (Hu et al., 2018), mammalian predators (Silver‐Gorges et al., 2021), domestic animals (Pheasey et al., 2018), and man‐made structures (Witherington et al., 2011) are not present on the Chandeleur Islands. This high level of protection paired with the remote location provides undisturbed sandy habitat for nesting sea turtles, which is becoming increasingly rare across the globe (Todd et al., 2019). One of the greatest threats to the Chandeleur Islands is from sea level rise (FitzGerald et al., 2016). Results of this study suggest loss of this habitat could negatively impact loggerhead and Kemp's ridley populations in the Gulf of Mexico.

Historically, loggerheads primarily used nesting beaches and in‐water foraging habitats in the eastern Gulf of Mexico (Foley et al., 2014; Hart et al., 2014), whereas Kemp's ridleys were tightly focused on nesting beaches in northern Mexico and southern Texas (Shaver et al., 2016). As their nesting ranges expand, potential for overlap, and as such interspecific competition, may increase and that overlap would most likely occur in eastern Louisiana and Mississippi (Hart et al., 2018). Although both sea turtle species are currently nesting on the Chandeleur Islands, nesting density and species phenology may limit interspecific competition, thereby allowing for greater range expansion by both species (Svenning et al., 2014). For example, in the Northern Gulf of Mexico, loggerheads nest at night primarily from late May to early August, whereas Kemp's ridleys nest during the day primarily from April to mid‐July (Shaver et al., 2016). The large size of the islands (i.e., amount of available nesting habitat) and these phenological differences may be enough to reduce competitive potential between the two species, thereby permitting increased range expansion.

Another issue that could arise from increased range overlap between loggerheads and Kemp's ridleys is the possibility of hybridization. Hybridization involves interbreeding of distinct evolutionary groups or species (Pfennig et al., 2016). It can increase genetic diversity and adaptive divergence (Abbott et al., 2013; Almeida et al., 2023) but also threaten small populations by reducing growth rate through production of nonviable offspring and dilute the genetic make‐up of species (Almeida et al., 2023; Todesco et al., 2016). Additionally, it may play an important role in shaping a species range (Pfennig et al., 2016). Hybridization has been documented among sea turtle species. For example, in Brazil, hybridization rates can reach up to 42% between hawksbills and loggerheads (Lara‐Ruiz et al., 2006). Although reported relatively infrequently, hybridization between loggerheads and Kemp's ridleys has been reported, including a first‐generation hybrid nesting female that deposited multiple, viable clutches in Georgia in multiple years (Pfaller et al., 2019, 2021).

The identification of previously understudied nesting sites can provide important information about sea turtle population status and trends (Flores et al., 2021; Wallace et al., 2011). For example, surveys of remote beaches along the coast of Panama resulted in discovery of a site that supported relatively large numbers of nesting Pacific green turtles (Flores et al., 2021). Demographic modeling that provides population‐level information relies on the most complete data available for accurate results. Gaps in data that remain when remote beaches are not surveyed can impact those modeling efforts (Pilcher & Chaloupka, 2013). The nest numbers documented in this study suggest that the development of a sea turtle monitoring program for the Chandeleur Islands, and in fact sandy beaches along the entire Louisiana coast (Hildebrand, 1979), could help improve management of both northern Gulf of Mexico loggerheads and endangered Kemp's ridleys.

## AUTHOR CONTRIBUTIONS

**Margaret M. Lamont:** Conceptualization (equal); formal analysis (lead); investigation (supporting); methodology (lead); visualization (equal); writing – original draft (lead); writing – review and editing (lead). **Dianne Ingram:** Conceptualization (equal); formal analysis (equal); investigation (equal); methodology (equal); visualization (equal); writing – original draft (supporting); writing – review and editing (supporting). **Todd Baker:** Conceptualization (lead); formal analysis (supporting); funding acquisition (lead); investigation (lead); project administration (lead); resources (lead); visualization (supporting); writing – review and editing (supporting). **Matt Weigel:** Conceptualization (supporting); formal analysis (supporting); funding acquisition (supporting); investigation (equal); project administration (equal); resources (equal); visualization (supporting); writing – review and editing (supporting). **Brian M. Shamblin:** Formal analysis (supporting); methodology (supporting); visualization (supporting); writing – review and editing (supporting).

## FUNDING INFORMATION

This project was conducted as part of the project titled Conservation and Enhancement of Nesting and Foraging Habitat for Birds, Component 1: Chandeluer Islands, LA, which was funded by the *Deepwater Horizon* Regionwide TIG through their Final Restoration Plan/Environmental Assessment 1: Birds, Marine Mammals, Oysters, and Sea Turtles (https://www.gulfspillrestoration.noaa.gov/sites/default/files/FINAL.RP_EA‐2021.09.16‐TIG.approved_0.pdf).

## CONFLICT OF INTEREST STATEMENT

The authors declare no conflicts of interest.

## Data Availability

All data, except location information, are presented in the manuscript. Because location information for imperiled species is sensitive and this project was conducted in a protected area, those data will not be made publicly available. Please contact the lead author for additional information.
